# Systolic Peak Detection in Acceleration Photoplethysmograms Measured from Emergency Responders in Tropical Conditions

**DOI:** 10.1371/journal.pone.0076585

**Published:** 2013-10-22

**Authors:** Mohamed Elgendi, Ian Norton, Matt Brearley, Derek Abbott, Dale Schuurmans

**Affiliations:** 1 Department of Computing Science, University of Alberta, Edmonton, Alberta, Canada; 2 National Critical Care and Trauma Response Centre, Darwin, Northern Territory, Australia; 3 School of Electrical and Electronic Engineering, University of Adelaide, Adelaide, South Australia, Australia; Georgia State University, United States of America

## Abstract

Photoplethysmogram (PPG) monitoring is not only essential for critically ill patients in hospitals or at home, but also for those undergoing exercise testing. However, processing PPG signals measured after exercise is challenging, especially if the environment is hot and humid. In this paper, we propose a novel algorithm that can detect systolic peaks under challenging conditions, as in the case of emergency responders in tropical conditions. Accurate systolic-peak detection is an important first step for the analysis of heart rate variability. Algorithms based on local maxima-minima, first-derivative, and slope sum are evaluated, and a new algorithm is introduced to improve the detection rate. With 40 healthy subjects, the new algorithm demonstrates the highest overall detection accuracy (99.84% sensitivity, 99.89% positive predictivity). Existing algorithms, such as Billauer's, Li's and Zong's, have comparable although lower accuracy. However, the proposed algorithm presents an advantage for real-time applications by avoiding human intervention in threshold determination. For best performance, we show that a combination of two event-related moving averages with an offset threshold has an advantage in detecting systolic peaks, even in heat-stressed PPG signals.

## Introduction

It has been shown that atherosclerosis, the underlying cause of coronary heart disease, can occur even in children and adolescents [1]–[3]. This fact supports the belief that primary prevention of atherosclerosis ought to commence in childhood. Monitoring arterial vascular walls as well as risk factors such as hypertension, hypercholesterolemia, and other blood biochemical profiles can potentially help identify individuals at increased risk of developing atherosclerosis in adulthood. Moreover, recent studies have shows that increased arterial stiffness can impact subsequent development of coronary heart disease [4]. Considering these facts, it is necessary to develop a simple and convenient screening method for the early identification of those people at risk, and those who have an additional stimulus of a heated environment.

**Figure 1:**
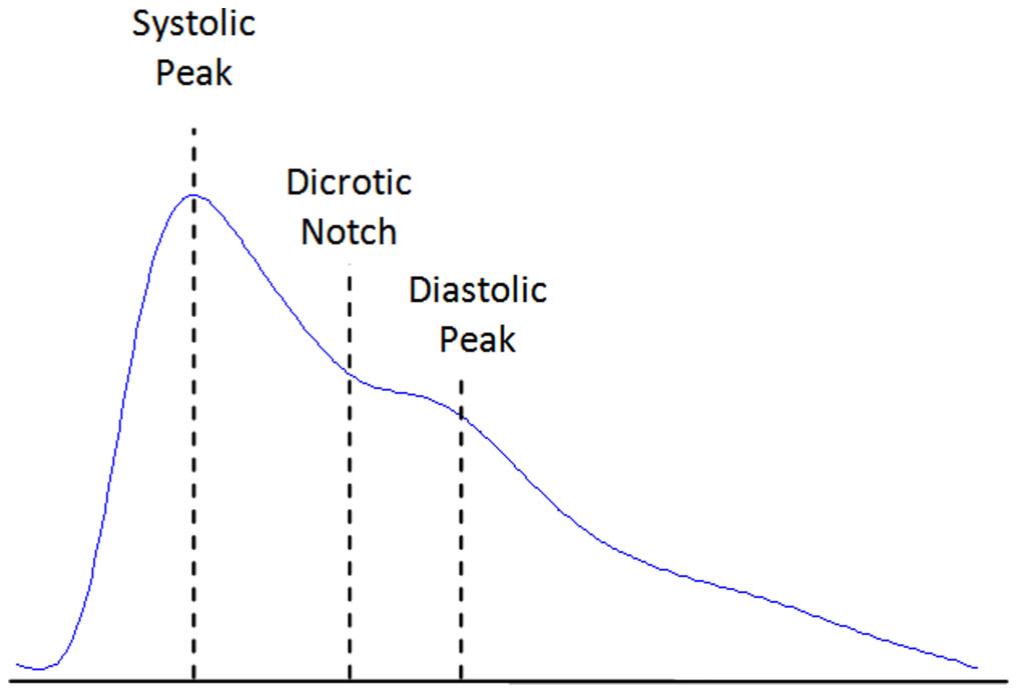
Fingertip photoplethysmogram signal measurement. The photoplethysmogram waveform consists of one systolic wave and one diastolic wave.

Pulse-wave analysis has been shown to provide valuable information on aortic stiffness and elasticity [5]–[7], and it has been widely used to evaluate the vascular effects of aging, hypertension and atherosclerosis [8]–[11]. Photoelectric plethysmography, a common method of pulse-wave analysis, has been referred to as photoplethysmography (PTG/PPG) and digital volume pulse (DVP) analysis; however, the acronym PPG will be used exclusively within this study according to recommendations in Ref. [12]. Fingertip photoplethysmography mainly reflects the pulsatile volume changes in the finger arterioles, as shown in Figure 1, and has been recognized as a noninvasive method for measuring arterial pulse waves in relation to changes in wave amplitude [13]. This convenient and objective technique for analyzing the PPG wave has now commonly replaced conventional methods. Several epidemiological studies have demonstrated that the information extracted from the PPG waveform is associated closely with age and other risk factors for atherosclerotic vascular disease [10], [14].

Although the clinical significance of PPG measurement has been well investigated, there remains a lack of research focusing on the automatic detection of systolic peaks in PPG signals. Therefore this investigation, the first of its kind, is aimed at evaluating a fast and robust algorithm for detecting systolic peaks in PPG signals under heat stress conditions. Heat-stress exercise changes the PPG waveform pattern and as a consequence affects the detection of systolic peaks. After severe exercise conditions, motion artifacts present a challenge for PPG analysis [15]. Therefore, in this study, we evaluate three well-known peak detection algorithms on PPG signals, measured at rest and after exercise in tropical conditions. Furthermore, we introduce a novel algorithm that uses two event-related moving averages to eliminate the need for human intervention for the determination of the patient-specific threshold. The final goal of this study is to determine the ideal algorithm for distinguishing irregular PPG patterns, especially after exercise, by quantitatively comparing the various systolic-peak detection methods and delineating where they fail.

## Materials and Methods

### Data Collection

**Figure 2:**
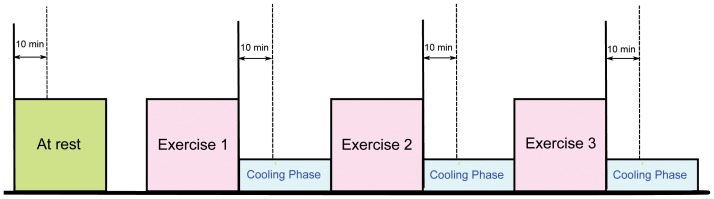
Measurement Protocol. The duration of the whole experiment was approximately 4

The heat stress PPG data for this study were collected as part of a National Critical Care and Trauma Response Centre (NCCTRC) project to assess the physiological and perceptual responses of emergency responders to a simulated chemical, biological, and radiological (CBR) incidents in tropical environmental conditions, for comparison of the effectiveness of various cooling methods. The background of the project can be found in Ref. [16]. Forty healthy, heat acclimatised emergency responders (30 males and 10 females) with a mean ± SD age of 34.7±6.6 volunteered and provided written informed consent to participate this study, which was approved by the Human Research Ethics Committee of the Northern Territory Department of Health and Menzies School of Health Research. Subjects undertook 30 minutes of triaging and resuscitating, transporting and decontaminating weighted manikins while wearing Level 3 PPE, which comprises a fully enclosed, impermeable suit including boots, gloves, hood, face mask and respirator (SE400i, S.E.A. Group, Warriewood, Australia) followed by 30 minutes of rest and cooling. This protocol was repeated three times with PPG data collected during each rest period as shown in Figure 2. Here, PPG data were measured by a photoplethysmography-equipped device (Salus APG, Japan) at a sampling rate of 367 Hz, with the sensor located at the cuticle of the second digit of the left hand. Measurements were taken for 20 seconds while subjects were undertaking seated rest. An emergency physician annotated the systolic peaks as controls for evaluation. The subjects were normotensive (mean systolic blood of 129.3 mmHg, range 110–165 mmHg), and had no known cardiovascular, neurological or respiratory disease. Prior to the experiment, the subjects provided information about their physical condition. Physical information such as height and weight were also measured for demographical research and summarized in Table 1. Every experiment was performed in a typical fire-fighting centre. Drinking and smoking were prohibited during 24 hrs and 2 hrs before experiment, respectively. For signal conditioning and peak detection, MATLAB 2010b (The MathWorks, Inc., Natick, MA, USA) was used. An Omron HEM-907 was used for blood pressure measurement.

**Table 1:** Demographic data for the subjects in this study.

Characteristic	Mean	Standard Deviation
Age (yrs)	34.7	6.6
Body Mass (kg)	81.8	12.8
Height (cm)	176.0	6.5
Body Mass Index (k*g*.m^−2^)	26.3	3.6
Resting Heart Rate (bpm)	76.0	14.7
Resting Systolic Blood Pressure (mmHg)	129.3	13.3

### Systolic Peak Detection Algorithms

We discuss and evaluate three algorithms that are commonly used in the real-time analysis of PPG signals, and introduce a new algorithm that demonstrates greater robustness and accuracy for systolic peak detection under conditions of heat stress. All of the algorithms we evaluate are advantageous in that they do not impose an extensive computational overhead, while avoiding manual segmentation and patient-specific modifications that are often required in biosignal analysis.

### Method I: Local Minima and Maxima

**Figure 3:**
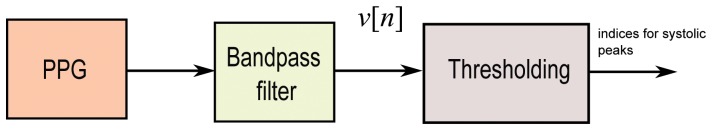
Flowchart for Method I. This systolic peak time-domain detection algorithm consists of two stages: bandpass filter and thresholding.

The idea of detecting peaks by finding the local maxima and minima in noisy signal has been investigated in several studies [17]–[21]. Billauer [22] developed an algorithm that detects peaks using the local maxima (peak) and minima (valley) values based on the architecture shown in Figure 3. Recently, Billauer's algorithm has been used to detect the respiratory and cardiac peaks [18]. Moreover, Domingues [23] used Billauer's algorithm to detect systolic peaks in PPG signals. A bandpass filter (0.5–8 Hz) was introduced to enhance the signal during the maxima (peak) search; however, a bandpass filter was not used in Billauer's implementation. For the thresholding operation, a point is considered a maximum peak if it has a maximal value and if the prior time-step possessed a value lower than 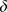,
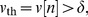
(1)where 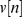 is the filtered PPG signal (cf. Figure 3) and 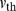 is filtered PPG signal after applying the threshold 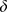. Here, 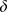 is a variable that is at least equal to the maximum difference of amplitude, which can be mistaken as a peak. In this investigation we set 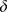 to 0.1.

### Method II: First Derivative with Adaptive Thresholds

**Figure 4:**
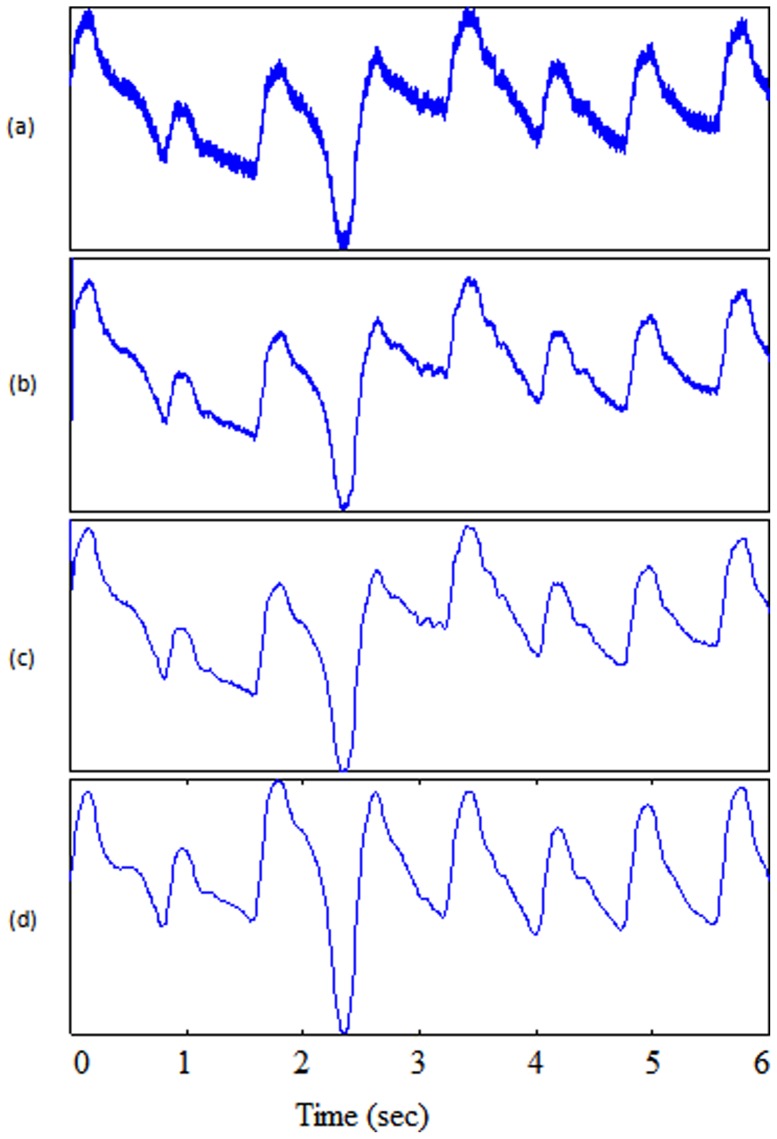
Filtering output. (a) Original signal PPG from record I-31 measured at rest (b) low-pass filter in Method II (c) low-pass filter in Method III (d) band-pass filter in Method VI.

**Figure 5:**
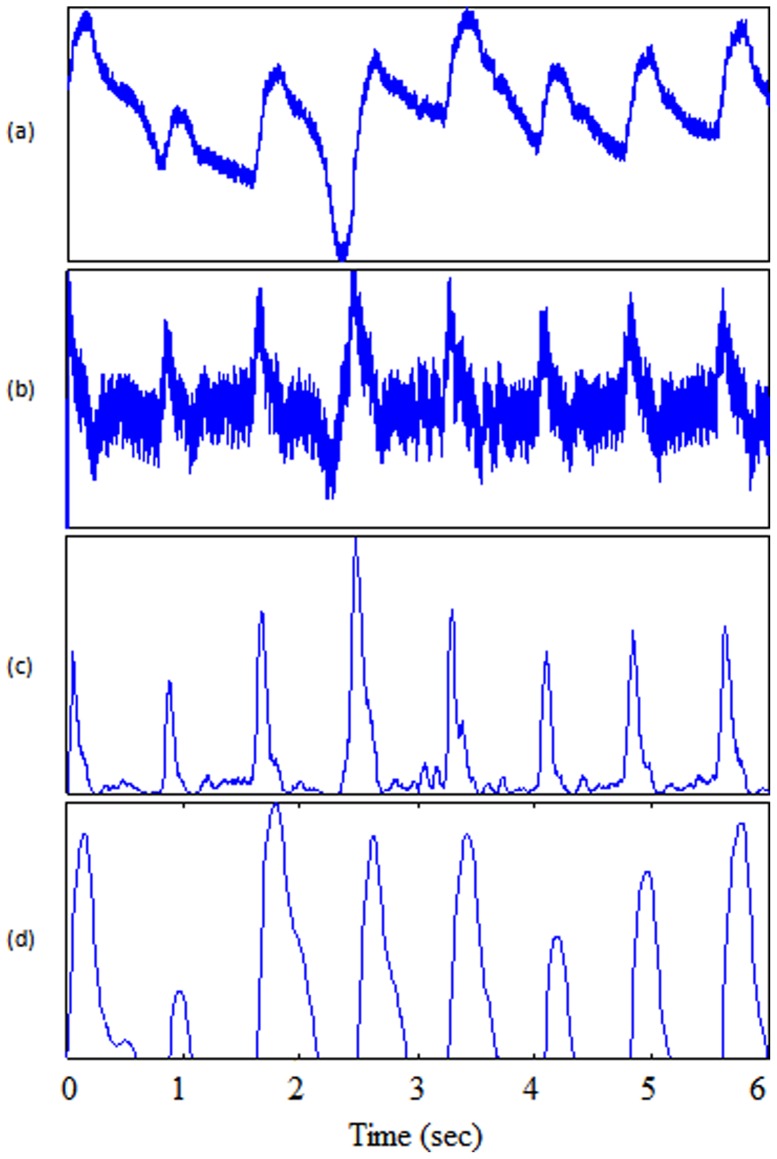
Features output. (a) Original signal PPG from record I-31 measured at rest (b) derivative calculation in Method II (c) slope-sum in Method III (d) squaring in Method VI.

**Figure 6:**
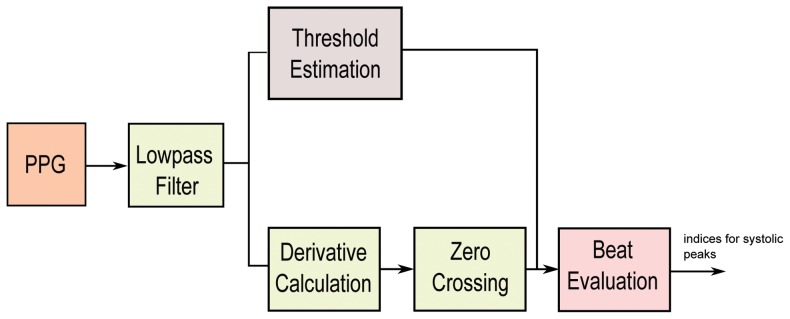
Flowchart for Method II. This systolic peak time-domain detection algorithm consists of three main stages: lowpass filter, slope sum function, and thresholding.

Usually, a band pass filter is initially applied to the raw PPG signal to suppress noise and artifacts. However, Li et al. [24] solely utilized a low pass filter, as shown in Figure 4(b), since they consider the derivative to be equivalent to a high-pass filter. The algorithm of Li et al. [24] applies a first derivative estimate to obtain zero-crossing points that are used to evaluate the inflection points in the original PPG signal. Using the derivative, as shown in Figure 5(b), the onset of a PPG waveform can be related to a zero-crossing point before a maximal inflection, while the systolic peak is related to a zero-crossing point after that inflection. Their delineator first seeks the candidate zero-crossing points in the derivative estimate. Nevertheless, various noise sources and artifacts often distort the raw PPG waveforms, thereby introducing many spurious points in the derivative estimates. The threshold estimation block, shown in Figure 6, indicates that the delineator has to estimate the amplitude and interval thresholds adaptively. It first segments the filtered PPG waveforms into multiple equal divisions (i.e., each division with equal waveform samples), and then applies a selective window (duration of 2 s) to the beginning of each division. Within those windows, the amplitudes and pulse rates are subsequently estimated and averaged as the initial thresholds. Then, the delineator searches for pairs of inflection and zero-crossing points in the PPG waveforms and their derivative in a beat-by-beat manner. Finally, within the beat evaluation phase, the delineator then re-examines the PPG waveforms, and identifies candidate onsets and systolic peaks based on both amplitude and interval thresholds determined in the threshold estimation phase.

### Method III: Slope Sum function with An Adaptive Threshold

**Figure 7:**
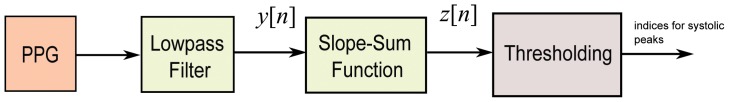
Flowchart for Method III. This systolic peak time-domain detection algorithm consists of three main stages: lowpass filter, slope sum function, and thresholding.

A low pass filter applied before the slope sum function, followed by adaptive thresholding, is used by Zong et al. [25]. The outputs of the PPG signal after applying the low pass filter and slope sum are shown in Figure 4(c) and Figure 5(c) respectively. The main purpose of the slope sum function is to enhance the upslope of the PPG pulse and to suppress the remainder of the pressure waveform. In particular, the slope sum is defined as
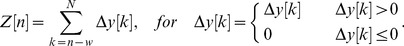
(2)Here 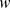 is the length of the analysing window, which Zong et al. [25] approximate to be equal to 128 ms or 47 samples for the sampling frequency (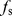) of 367 Hz; the index 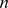 ranges over 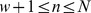 where 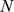 is the total number of PPG samples in the record; and 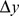 is 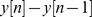, where 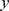 is a filtered PPG signal shown in Figure 5(c). As can be seen Figure 7, the final thresholding stage is conducted adaptively. First, the adaptive threshold is initialized to three times the mean signal 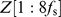, averaged over the first eight seconds of the recording, as follows:
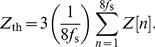
(3)


The threshold base value is adaptively updated by the maximum 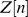 value for each pulse detected. The actual threshold is taken to be 60% of the threshold base value, as suggested by [25]. When 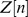 crosses this threshold, the algorithm searches for the minimum and maximum 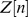 values in a 150 ms window preceding and following the threshold-crossing point, respectively. The pulse detection is accepted only if the difference between the maximum and minimum exceeds a certain value; otherwise the pulse detection is rejected. When the pulse is accepted, the algorithm searches backward in time from the threshold-crossing point for the onset of the 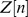 pulse. The onset point is determined when the slope sum signal exceeds 1.0% of the maximum 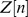 value. The calculated PPG onset is adjusted by 20 ms, to compensate for the low-pass filter's phase shift. Finally, to avoid double detection of the same pulse, a 300 ms eye closing (refractory) period is applied, during which no new pulse detection is initiated.

### Method IV: Event-Related Moving Averages with Dynamic Threshold

**Figure 8:**
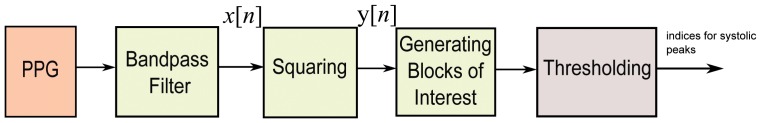
Flowchart for Method VI. This systolic peak time-domain detection algorithm consists of three main stages: pre-processing (bandpass filter), feature extraction (two moving averages), and classification (threshold).

In this study, we evaluate a novel algorithm adapted from the framework proposed by Elgendi for detecting 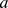 waves in APG (acceleration of PPG) signals [26]–[29] and for detecting QRS complexes in ECG signals [30]–[32]. The same approach will be used here to detect the systolic peaks. The method consists of three main stages: pre-processing (bandpass filtering and squaring), feature extraction (generating potential blocks using two moving averages) and classification (thresholding). The structure of the algorithm is given in Figure 8.

#### Bandpass Filter

A zero-phase second-order Butterworth filter, with bandpass 0.5–8 Hz, is implemented to remove the baseline wander and high frequencies that do not contribute to the systolic peaks [33]. The output of the zero-phase Butterworth filter applied to the PPG signal will be produce a filtered signal 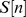, as shown in Figure 4(d). Then, clipping the output by keeping the signal above zero will produce signal 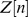.

#### Squaring

Squaring emphasises the large differences resulting from the systolic wave, which suppressing the small differences arising from the diastolic wave and noise, as shown in Figure 5(d). This step results in the output
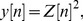
(4)which is important for improving the accuracy in distinguishing the systolic wave segment in PPG signals.

#### Generating Blocks of Interest

Blocks of interest are generated using two event-related moving averages that demarcate the systolic and heartbeat areas. The particular method used to generate blocks of interest has been mathematically shown to detect 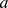 waves [27] and QRS complexes [30].

**Figure 9:**
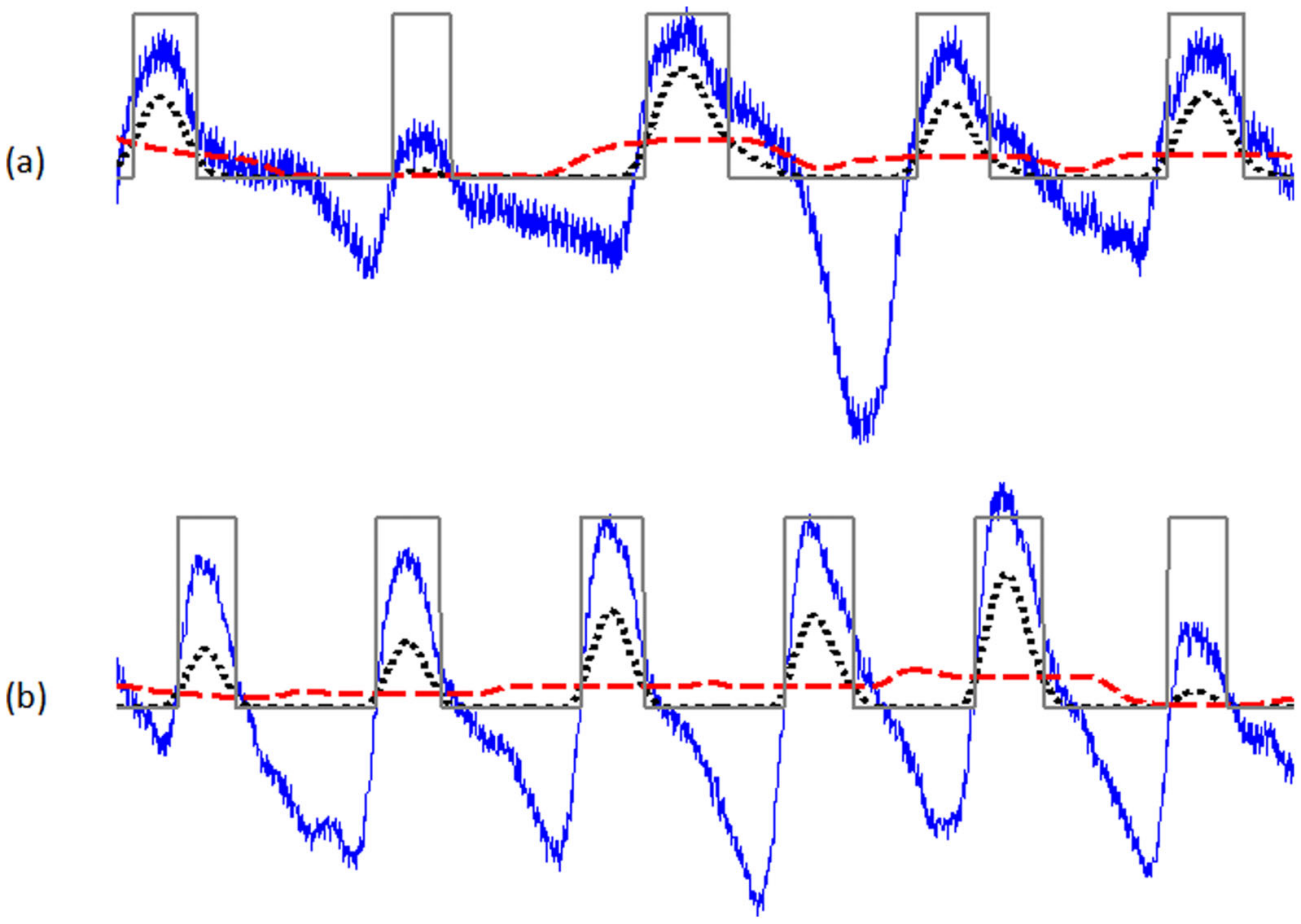
Generating blocks of interest using two moving averages. (a) Measured at rest I-31 (b) measured II-51 after exercise 1. The dotted signal (in black) is the first moving average 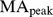 while the dashed signal is the second moving average 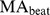. The squares are the blocks of interest.

In this procedure, the first moving average (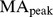) is used to emphasise the systolic peak area, as the dotted signal shown in Figure 9, and is given by
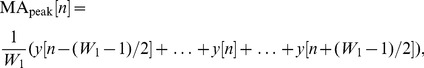
(5)where 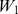 represents the window size of the systolic-peak duration. The resulting value is rounded to the nearest odd integer. The exact value for 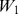 of 111 ms is determined after a brute force search, which will be discussed later in the parameter optimization section.

The second moving average (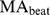) is used to emphasise the beat area to be used as a threshold for the first moving average, shown as a dashed signal Figure 9, and is given by
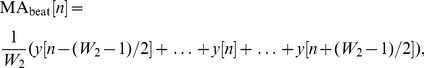
(6)where 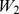 represents the window size of approximately one beat duration. Its value is rounded to the nearest odd integer. The exact value for 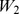 of 667 ms is determined after a brute force search, which will be discussed later in the parameter optimization section.

#### Thresholding

The equation that determines the offset level (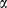) is 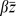, where 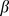 = 0.02 based on a brute force search that will be discussed later in the parameter optimization section, while 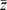 is the statistical mean of the squared filtered PPG signal. The first dynamic threshold value is calculated by shifting the 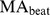 signal with an offset level 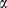, as follows:
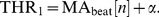
(7)


In this stage, the blocks of interest are generated by comparing the 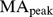 signal with 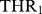, in accordance with the lines 9–16 shown in the pseudocode of Algorithm IV. Many blocks of interest will be generated, some of which will contain the PPG feature (systolic peak) and others will contain primarily noise. Therefore, the next step is to reject blocks that result from noise. Rejection is based on the anticipated systolic-peak width. In this paper, the undesired blocks are rejected using a threshold called 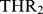, which rejects the blocks that contain diastolic wave and noise. By applying the 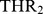 threshold, the accepted blocks will contain systolic peaks only,
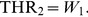
(8)


As discussed, the threshold 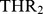 corresponds to the anticipated systolic wave duration. If a block is wider than or equal to 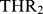, it is classified as a systolic peak. If not, it will be classified as noise. The last stage is to find the maximum absolute value within each block to detect the systolic peak; the code lines of this step are lines 18–25 in the pseudocode of Algorithm IV. Consecutive systolic waves are shown in Figure 9 to demonstrate the idea of using two moving averages to generate blocks of interest. Not all of the blocks contain potential systolic peaks; some blocks are caused by noise and need to be eliminated. Blocks that are smaller than the expected width for the systolic peak duration are rejected. The rejected blocks are considered to be noisy blocks and the accepted blocks are considered to contain a systolic peak. The detected systolic peaks are compared to the annotated systolic peaks to determine whether they were detected correctly. The search range for the true systolic peak is fixed to ±50 ms for all algorithms, to ensure consistency of comparison. (Table 2. Algorithm IV)

**Table 2:** Algorithm IV: Detector (PPG _signal_, *F*_1_, *F*_2_, *W*_1_, *W*_2_, *β*

1: *S*_ peaks ←_ {}
2: Filtered = Bandpass(PPG_signal_, *F*_1_–*F*_2_)
3: Clipped = Clip(Filtered)
4: Q _clipped_ = Square(Clipped)
5: MA_peak_ = MA(Q_ clipped_,*W*_1_)
6: MA_beat_ = MA(Q _clipped_,W_2_)
7: 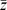 = mean(Q _clipped_)
8: 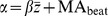
9: THR_1_ = MA_beat_+α
10: **for** n = 1 **to** length(M_Apeak_) **do**
11: **if** MA_peak_[n]>THR_1_ **then**
12: BlocksOfInterest[n] = 0.1
13: **else**
14: BlocksOfInterest[n] = 0
15: **end if**
16: **end for**
17: Blocks←onset and offset from BlocksOfInterest
18: **set** THR_2_ = *W*_1_
19: **for** j = 1 **to** number of Blocks **do**
20: **if** width(Blocks[j]) ≥ THR_2_ **then**
21: S _peaks_←index of max. value within the block
22: **else**
23: ignore block
24: **end if**
25: **end for**
26: **Return** (*S*_peaks)_

#### Parameter Optimization

Performance of systolic peak detection algorithms is typically evaluated using two statistical measures: 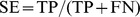 and 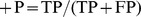, where TP is the number of true positives (systolic peak detected as systolic peak), FN is the number of false negatives (systolic peak has not been detected), and FP is the number of false positives (non-systolic peak detected as systolic peak). The sensitivity SE reports the percentage of true beats that were correctly detected by the algorithm. The positive predictivity +P reports the percentage of beat detections that were true beats. The function of the systolic peak detector (cf. pseudocode of Algorithm IV) has five inputs: the PPG signal (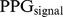), frequency band (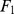
–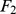), event-related durations 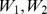, and the offset (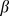). Any change in these parameters will affect the overall performance of the proposed algorithm. These parameters are interrelated and cannot be optimised in isolation. A rigorous optimization via brute-force search, over all parameters, is conducted (cf. Table 3). This is a time consuming process, but required before making definitive claims. The data used in this training phase are the PPG signals measured at rest. Optimization of the beat detector's spectral window for the lower frequency resulted in a value within 0.5–1 Hz with the higher frequency within 7–15 Hz. The window size of the first moving average (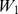) varies from 54 ms to 111 ms, whereas the window size of the second moving average (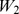) varies from 545 ms to 694 ms. The offset 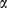 was tested over the range 0–10% of the mean value of the squared filtered PPG signal. The QRS complex corresponds roughly to the systolic duration in PPG, which is 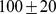 ms in healthy adults [34]. Interestingly, the algorithm uses an optimal value of 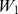 (111 ms) corresponding to the systolic peak duration, and an optimal value of 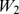 (667 ms) for the heartbeat duration. It is clear from Table 3 that the optimal frequency range for the systolic detection algorithm over the database is 0.5–8 Hz. Moreover, the optimal values for the moving-average window sizes and offset are 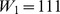 ms, 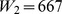 ms, and 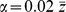. The systolic algorithm is adjusted with these optimal parameters. Then, the detector is tested on three PPG after-exercise measurements without any further adjustment.

**Table 3:** A rigorous optimisation over all parameters of the systolic peak detection algorithm: frequency band, 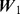, 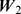, and the offset 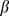.

Iterations	Frequency Band	*W*_1_	*W*_2_	*β*	SE	+P	Overall Accuracy (%)
1	0.5–8 Hz	111	667	2	100.00	99.72	99.86
2	0.5–8 Hz	111	667	3	100.00	99.72	99.86
3	0.5–8 Hz	111	694	2	100.00	99.72	99.86
4	0.5–8 Hz	111	694	3	100.00	99.72	99.86
5	0.5–9 Hz	111	667	2	100.00	99.72	99.86
6	0.5–9 Hz	111	694	2	100.00	99.72	99.86
7	0.5–9 Hz	111	694	3	100.00	99.72	99.86
8	0.5–7 Hz	111	694	2	99.90	99.72	99.81
9	0.5–7 Hz	69	694	9	99.90	99.72	99.81
10	0.5–7 Hz	69	694	10	99.90	99.72	99.81
11	0.5–7 Hz	83	639	10	99.90	99.72	99.81
12	0.5–7 Hz	83	667	8	99.90	99.72	99.81
13	0.5–7 Hz	83	667	9	99.90	99.72	99.81
14	0.5–7 Hz	83	667	10	99.90	99.72	99.81
15	0.5–7 Hz	83	694	6	99.90	99.72	99.81
16	0.5–7 Hz	83	694	7	99.90	99.72	99.81
17	0.5–7 Hz	83	694	8	99.90	99.72	99.81
18	0.5–7 Hz	83	694	9	99.90	99.72	99.81
19	0.5–7 Hz	83	694	10	99.90	99.72	99.81
20	0.5–7 Hz	97	611	8	99.90	99.72	99.81
.	.	.	.	.	.	.	.
.	.	.	.	.	.	.	.
.	.	.	.	.	.	.	.
.	.	.	.	.	.	.	.
5606	1–15 Hz	69	556	0	98.27	95.78	97.02
5607	1–12 Hz	56	556	0	98.27	95.67	96.97
5608	1–13 Hz	56	556	0	98.27	95.57	96.92
5609	1–14 Hz	56	556	0	98.27	95.57	96.92
5610	1–15 Hz	56	556	0	98.27	95.57	96.92

All possible combinations of parameters (5,610 iterations) have been investigated and sorted in descending order according to their overall accuracy. The data used in this training phase was PPG measured at rest. The overall accuracy is the average value of SE and +P.

## Results and Discussion

**Figure 10:**
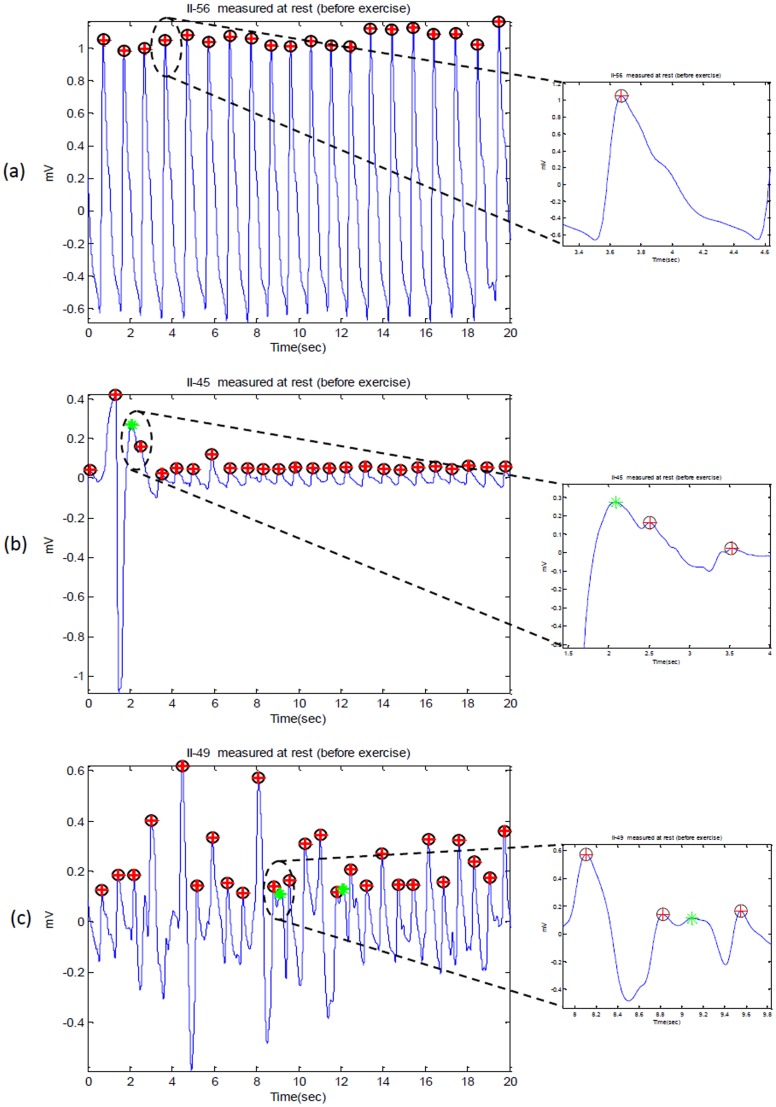
Detected systolic peaks in PPG signals at rest (before exercise). Here, ‘O’ represents the annotated systolic peak, ‘+’ represents detected peak by the algorithm, while ‘*’ represents the false positive.

**Figure 11:**
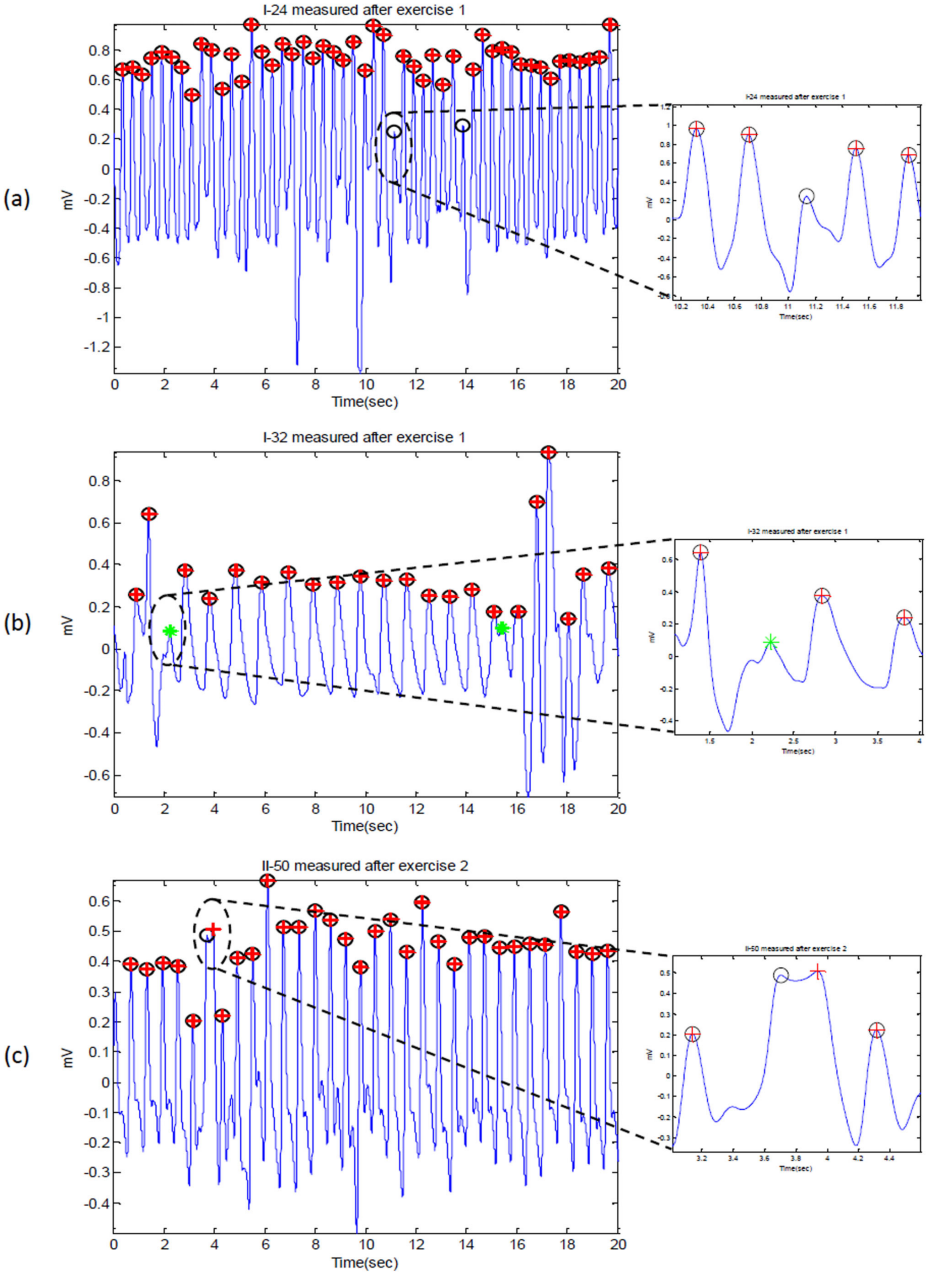
Detected systolic peaks in PPG signals after exercise. Here, ‘O’ represents the annotated systolic peak, ‘+’ represents detected peak by the algorithm; thus, when the circle has red-cross inside, it means the annotated peak has been correctly detected. If the circle is empty it means false negative; while ‘*’ represents the false positive.

The algorithms were tested on 40 subjects with PPG signals measured at four time points: before exercise, after exercise 1, after exercise 2, and after exercise 3; with total number of 160 recordings. The main objective is to evaluate the robustness of the algorithms against the non-stationary effects, low SNR, and high heart rate exhibited after exercise in conditions of heat stress. Under controlled conditions (e.g. hospital and clinic), analyzing stationary PPG signals is straightforward; as systolic peaks have similar amplitudes, the statistical characteristics of the signals (i.e. mean and standard deviation) do not change appreciably with time, and a simple threshold level can effectively detect systolic peaks. Figures 10(a) and 11(a) represent the PPG signals with stationarity effects for volunteer I2 (before exercise) and G2 (after exercise) (all systolic peaks are almost straight-lined). By contrast, our heat-stress study was performed in a typical fire-fighting centre, PPG signals become non-stationary, which makes analysis difficult since the standard deviation changes with time (systolic peak amplitudes vary with time and simple level thresholds cannot optimally detect systolic peaks). This has a negative effect on detection algorithm performance, especially PPG signal collected at rest. PPG signals collected after exercises suffered from low and high frequencies because of the sweat and exhaustion of the volunteer; however, the bandpass filter succeeded in removing these artifacts. It is worth noting that the detection accuracy of any algorithm will increase for after exercise PPG measurement. This is because of the fast rhythm caused by the stress test, which decreases the time duration between two consequent heart pulses and emphasizes the systolic peak in PPG wave form (cf. Figure 9).

Table 4 demonstrates the performance of the described algorithms on the data collected under various conditions. It also shows the advantages of each algorithm through true positives and disadvantages through false positives and false negatives. Method I introduced by Billauer, which detects peaks in any signal based on local minima-maxima, provides a baseline detection rate. However, it is not an optimal methodology for detecting systolic peaks in PPG signals under varying conditions. In a previous study, Method II achieved a SE of 99.88% and a +P of 98.69% in detecting systolic peaks over 2,564 beats [24]. However, its performance in the current study, conducted on 5,071 beats, resulted in a lower SE of 97.9% and a +P of 99.93%. The main difference in the present study is that the PPG recordings contain sudden amplitude drops and low signal-to-noise ratios due to the more stressful conditions under which the signals were captured. In this case, Method II was unable to control the number of FPs, consequently +P decreased. Method III generally incurred the same set of errors as Method II in our study, but with additional FNs. Note that in a previous study, Method III has been applied on 39,848 beats and obtained an impressive SE of 99.69% and a +P of 99.71% in detecting PPG beats not systolic peaks [25]. The reason for the reduced performance of Method III in our study appears to be arrhythmia: the sudden decrease in amplitudes and low signal-to-noise ratios among the PPG signals we collected.

**Table 4:** Systolic peak detection performance on PPG-CBR responders in tropical condition database.

Test	Algorithm	FP	FN	SE (%)	+P (%)
Before Exercise	Method I	26	33	96.55	97.66
	Method II	0	36	96.06	100.00
	Method III	23	42	95.50	97.88
	Method VI	3	0	100.00	99.72
After Exercise 1	Method I	13	27	98.07	99.00
	Method II	1	15	98.67	99.85
	Method III	17	39	96.90	98.67
	Method VI	2	3	99.83	99.79
After Exercise 2	Method I	45	13	98.86	97.29
	Method II	1	26	97.89	99.89
	Method III	7	43	96.71	99.48
	Method VI	1	2	99.86	99.92
After Exercise 3	Method I	36	20	98.19	98.02
	Method II	0	11	99.09	100.00
	Method III	3	37	97.26	99.76
	Method VI	1	2	99.86	99.91

The PPG signals were collected from 40 heat acclimatised emergency responders for 20 seconds during the 10 minutes break of each exercise (cf. Figure 2). To evaluate the performance of each algorithm in each test, two statistical measures are used: 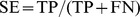 and 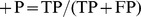, where TP is the number of true positives (systolic peak detected as systolic peak), FN is the number of false negatives (systolic peak has not been detected), and FP is the number of false positives (non-systolic peak detected as systolic peak).

The proposed algorithm (Method VI) scored the highest sensitivity and positive predictivity rates among the four algorithms. The proposed algorithm appears to be more robust against effects of post-exercise measurement non-stationarity during hot/humid conditions. The results show that the proposed Method IV is able to detect systolic peaks correctly in non-stationary PPG signals before exercise, as shown in Figure 10(a). However, in case of non-stationary PPG signals produced after exercise, the algorithm did incur a few instances of failure, as shown in Figures 10(b,c) and Figure 11. The cause of the failure is due to the extremely low amplitude systolic waves in heat-stressed PPG signals (cf. Figures 10 and 11). In such cases, applying a simple level threshold is not an effective approach. The proposed Method IV, however, handles varying amplitudes well compared to the other three algorithms. In fact, it is clear that the proposed algorithm is more amplitude-independent and is able to detect the systolic peaks in various voltage range.

The analysis of a regular heart rhythm is simple, as the systolic peaks are repeated with an equally spaced pattern. This regularity helps the time-domain threshold methodologies to detect systolic peaks successfully. The regular heart rhythm is called the normal sinus rhythm in PPG signals [35], which means the rhythm is constant and the occurrence of the next beat is predictable. The proposed algorithm easily detects systolic peaks correctly in PPG signals with a regular heart rhythm as shown in Figure 10(a). The sensation of an irregular heart rhythm is usually related to either premature beats or atrial fibrillation.The proposed algorithm performed relatively better than the other algorithms in detecting systolic peaks with premature beats in both conditions at rest and after exercise.

All of the algorithms failed on specific instances in the PPG recordings. Possible reasons for failures included the following:

1. False-positive detection of systolic peak because of
  - Arrhythmia existence (i.e. premature ventricular contractions)
  - Wide beat duration
2. False-negative detection due to
  - Sudden drop in amplitude
  - Low signal-to-noise ratio.

Although the duration of the systolic peaks changed dramatically after exercise, the proposed Method IV succeeded in detecting the systolic peaks effectively. As discussed above, the proposed method successfully detected systolic peaks in PPG signals with a low SNR, non-stationarity, irregular heart rhythms, and before and after exercise. Figures 10(b,c) and 11(b) demonstrate the false positives caused by arrhythmia; while Figures 11(a,c) demonstrate the false negatives caused by sudden drop in amplitude.

### Limitations of Study and Future Work

One of our next steps regarding the result of this study is to examine the correlation of the accurately detected heart rates using PPG signals with age, BMI, and core temperature.

It is important to note that the number of PPG records (total of 40) for testing is modest. A larger sample size and a more diverse data set are needed in order to generalise the findings of this study. Evaluation of systolic-peak detection was challenging in this study because the number of annotated beats did not allow all possible morphologies found in PPG signals under conditions of heat stress to be well represented. To our knowledge, there is no available PPG database measured in tropical conditions or after heat stress that would allow a more thorough assessment and comparison of the tested algorithms.

In future studies it may be advisable to have multiple PPG systems to collect the signal immediately after exercise. In the present study, data were collected immediately after exercise, however, some subjects may have cooled down whilst queuing for measurement.

Technically, exploring the event-related moving average methodology for detecting events in PPG signal is promising in terms of computational complexity and efficiency. This can be further improved by investigating other bandpass filters, with different orders, and also by developing fast moving average techniques for real-time analysis and mobile phone applications.

## Conclusion

For all the algorithms, the detection errors arose from a variety of factors including the existence of wide premature ventricular contractions, low-amplitude peaks, peaks with reversed polarity, and signals with low SNR. The application of an event-related dual moving average allows the accurate, computationally simple algorithm we propose to be used for real-time applications and the processing of large databases. A detection algorithm for systolic peaks in PPG signals measured from medical responders in a tropical environment has not been previously addressed in the literature. However, we have demonstrated that a robust algorithm can be developed for detecting systolic peak in PPG signals collected in a hot environment with high-frequency noise, low amplitude, non-stationary effects, irregular heartbeats, and high heart rates. The algorithm was evaluated using 40 records, containing 5,071 heartbeats, with an overall sensitivity of 99.89% and the positive predictivity was 99.84%.
